# The performance of microbial fuel cell with sodium alginate and super activated carbon composite gel modified anode

**DOI:** 10.1186/s13568-024-01723-2

**Published:** 2024-06-06

**Authors:** Liangyue Cheng, Limin Jiang, Xiaowen Yang, Yuhao Gao, Ruiyuan Gai, Mingpeng Wang, Lei Chen

**Affiliations:** 1 School of Life Science, Qufu Normal University, 57 Jingxuan West Road, 273165 Qufu, Shandong People’s Republic of China; 2 Tianjin Institute of Industrial Biotechnology, Chinese Academy of Sciences, Tianjin Airport Economic Area, 32 West 7th Avenue, 300308 Tianjin, People’s Republic of China

**Keywords:** Anode modification, Sodium alginate, Super activated carbon

## Abstract

Microbial fuel cells (MFCs) have the functions of wastewater treatment and power generation. The incorporation of modified anodes enhances the sustainable power generation performance of MFCs. In this study, to evaluate the feasibility of sodium alginate (SA) as a biocompatible binder, hydrogel mixed with super activated carbon (SAC) and SA was modified the carbon cloth anode of MFC. The results showed that the maximum output voltage in the SAC/SA hydrogel modified anode MFC was 0.028 V, which was increased by 115%, compared with the blank carbon cloth anode. The internal resistance of MFC was 9429 Ω, which was 18% lower than that of control (11560 Ω). The maximum power density was 6.14 mW/m^2^, which was increased by 365% compared to the control. After modification of SAC/SA hydrogel, the chemical oxygen demand (COD) removal efficiency reached to 56.36% and was 12.72% higher than the control. Coulombic efficiency with modified anode MFC reached 17.65%, which was increased by 104%, compared with the control. Our findings demonstrate the feasibility of utilizing SA as a biocompatible binder for anode modification, thereby imparting sustainable and enhanced power generation performance to MFCs. This study presented a new selectivity for harnessing algal bioresources and improving anode binders in future MFC applications.

## Introduction

Microbial fuel cell (MFC) is a device capable of converting chemical energy into electrical energy from the early 1990s (Allen and Bennetto 1993). However, due to the low power output of MFCs, the further development and large-scale application of MFCs have been restricted (Logan and Regan 2006).

The anode usually acted as a carrier for the growth of microorganisms in a MFC (Sun et al. 2020). Its stability, biocompatibility, conductivity and active surface area directly affected the ability of microbial extracellular electrons transfer and the performance of MFCs (Liu et al. 2012). Many anode modification methods were performed to provide a larger electrocatalytic active area, more electrode surface charge or faster electron transfer rate of MFCs (Mateo et al. 2018), including: (i) Noble metals decoration. When carbon paper anode was decorated with the Au, total charge was increased in MFC inoculated with *Shewanella oneidensis* MR-1 (Sun et al. 2010). RuO_2_, which had a higher mass specific capacitance, excellent conductivity, a wider potential window and a high degree of redox reversibility (Lv et al. 2012). RuO_2_ modification could also increase power generation of the anodes (Li et al. 2018). Using sputtering technology to deposit Co on carbon paper significantly improved the adhesion density of *Saccharomyces cerevisiae* to it and the performance of yeast-based MFC (Kasem et al. 2013). (ii) High temperature treatment. When the graphite electrode was baked at 1100 ℃ in a kiln, the power density of the reactor was increased (Park and Zeikus 2002). The carbon cloth was heated at 450 °C for 30 min in air in a muffle furnace, maximum power density also increased (Cai et al. 2013). (iii) Modification of exogenous mediators. After graphite electrodes was modified with anthraquinone-1,6-disulfuric acid (AQDS), the faster reaction kinetics in MFC was obtained (Lowy and Tender 2008; Zhang et al. 2014). However, the above anode modification methods are expensive and complicated for large-scale application MFC. Thus, a cheap, simple, and bio-inclusive method is urgently needed to improve the performance of MFCs.

The adhesion between modified materials and electrodes is a crucial factor for anode modification aimed at enhancing MFC performance (Park et al. 2011). Sodium alginate [(C_6_H_7_O_6_Na)_n_] (SA) was a linear anionic polysaccharide extracted from brown algae (Gad et al. 2011). As a natural biopolymer, alginate had the advantages of good biocompatibility, biodegradability, non-toxicity (Sari-Chmayssem et al. 2015). The key characteristic of SA lies in its capacity to form gels when exposed to polyvalent metal ions, such as calcium (Anisha and Prema 2008). It was mainly composed of β-d-1,4-mannuronicacid (M) and α-l-1,4-guluronic acid (G) (Salomonsen et al. 2008). The homopolymeric regions of M residues and G residues (M and G blocks) were interspersed with regions of alternating structure (MG blocks) (Sriamornsak et al. 2007). The composition, sequence, and molecular weight of these residues contribute significantly to the wide variation in alginate’s physical properties (Gad et al. 2011). The application of the hydrogel as a binder was constantly being explored in recent years (Zhang et al. 2021). Biocompatible polyaniline-sodium alginate (Pani-SA) composites prepared on 3D porous nitrogen-doped carbon nanotubes/sponges (NCNT/S) were highly gridded, which had a larger specific surface area and active site than pure PANI, providing a larger area for bacterial adhesion and facilitating charge and ion transfer (Wang et al. 2020).

Super activated carbon (SAC) had the characteristics of good electrochemical performance and large capacity (Wang et al. 2016), usually with a specific surface area greater than 2000 m^2^/g due to its mesopores and micropores (Lin et al. 2015). The commercial SAC used in this study had been shown to have a specific surface area of 2500 m^2^/g (Yang et al. 2014). Therefore, SAC was chosen to be mixed with SA hydrogel to modify the anode of MFC. The impact of modification on the sustainable operation and electrochemical performance of the MFCs was assessed.

## Materials and methods

### Anodes preparation

Carbon cloths (projected area of 1 × 2 cm^2^, CeTech Co., Ltd, China) were used as work electrodes in all the MFCs. A total of 5 ml of 5% SA solution and 0.2 g SAC (YEC-8, Fuzhou Yihuan Carbon., Ltd, China) were mixed. The mixture 1 ml was dripped onto the carbon cloth anodes in experimental MFCs. Then, the surface was treated with a gradual addition of 50 ul of a CaCl2 solution (0.2 g/l) to form a gel. In control MFCs, carbon cloth without any modification was used as work electrode.

### Air-cathode preparation

The preparation of the cathode (7 cm^2^, 0.5 mm thick) referred to previous research (Dong 2013), the diffusion layer of the cathode was made of 60 mesh stainless steel mesh (SSM), a mixture of polytetrafluoroethylene (PTFE) (PTFE 60%, DAIKIN, Japan) and activated carbon (AC) (SUPER P Li, China). The catalytic layer of the cathode was loaded with 37.5 mg/cm^2^ of SAC as a catalyst. The ratio of the diffusion layer AC to PTFE was 1.5. The catalyst-coated layer of the cathode faced the anode. The AC and PTFE mixture coated layer faced the air.

### MFCs start-up and operation

The air-cathode single-chamber MFC with an volume of 28 ml was used in this study (Huang et al. 2016). Keep a distance of 1.5 cm between the two electrodes. The MFC was initially inoculated with pre-acclimated *Shewanella oneidensis* MR-1 (Our lab’s collection) suspensions from well-operated MFCs in the laboratory. The inoculum volume accounted for 14% of the reactor volume (V/V). The culture medium was M9 buffer (17.8 g/l Na_2_HPO_4_·12H_2_O, 3 g/l KH_2_PO_4_, 0.5 g/l NaCl, 1 g/l NH_4_Cl, 20.01 g/l CaCl_2_, 0.12 g/l MgSO_4_, 0.25 g/l yeast extract, 0.5 g/l tryptone, 0.5 g/l NaCl) (Zhang et al. 2014). Lactate was added to the anode cell to a final concentration of 18 mmol/l. The MFCs were purged with nitrogen gas for 30 min to make the system in anaerobic conditions. An external resistance of 1000 Ω was connected to the anode and cathode of the MFC reactor. When the output voltage of the MFC reactor dropped below 5 mV, the electrode solution in the MFC reactor was replaced. The reactor was operated in batch mode and the temperature was maintained at 30 ± 1 °C referring to previous work (Huang et al. 2016).The experiment was performed three times, and the optimal results were taken to be displayed.

### Morphology observation by a scanning electron microscope (SEM)

In this study, scanning electron microscope (SEM, Sigma 500 VP, Germany) was used to observe the surface morphology of anode materials, and to analyze the effect of anode surface morphology on the growth of electroactive microorganisms. A small piece of anode material was cut out and fixed on the stage with conductive glue, and then put into the SEM vacuum chamber for observation (Zhang 2020).

### Analysis and calculations

A data acquisition unit (T9357-3, Beijing Tiangong Wuhua Electromechanical Technology Co., Ltd.) was used to automatically record the MFC external circuit voltage. After MFCs had been running stably for 150 h, the polarization curves and power density curves were obtained by changing the external resistance in the range of 500–9500 Ω (Li et al. 2020). The current was calculated using Ohm’s law: $I=U/ R.$ The power density (*P*) of the anodes of the control and experimental groups in the MFCs were calculated according to equation $P=UI/A,$ where $U$ represents voltage, *I* represents the current with respect to applied voltage and *A* represents the area of the anode (2 cm^2^).

CE were calculated based on Eq. (1) (Oh et al. 2004).

1 $$\text{CE}=\frac{{C}_{Ex}}{{C}_{Th}}\times 100 {\%}$$

where *C*_*Ex*_ is the total coulombs calculated by integrating the current measured at each time interval (*i*) over time as ${C}_{Ex}=\sum _{i=1}^{t}\left({U}_{i}{t}_{i}\right)/R.$ Among them, ${U}_{i}$ denotes voltage at time interval *i.*${t}_{i}$ denotes time at time interval *i*. *C*_*Th*_, the theoretical value of coulombs that is obtained from lactate oxidation, was calculated as *C*_*Th*_*=FbMV*, where *F* is Faraday’s constant (96 485 C/mol of e^−^), *b* is number of moles of e^−^ produced from 1 mol of substrate, M is the lactate concentration (mol/l), and *V* is the liquid volume (L). Chemical oxygen demand (COD) was determined using the rapid digestion method (Yang 1998).

## Result

### Anode surface morphology

**Fig. 1:**
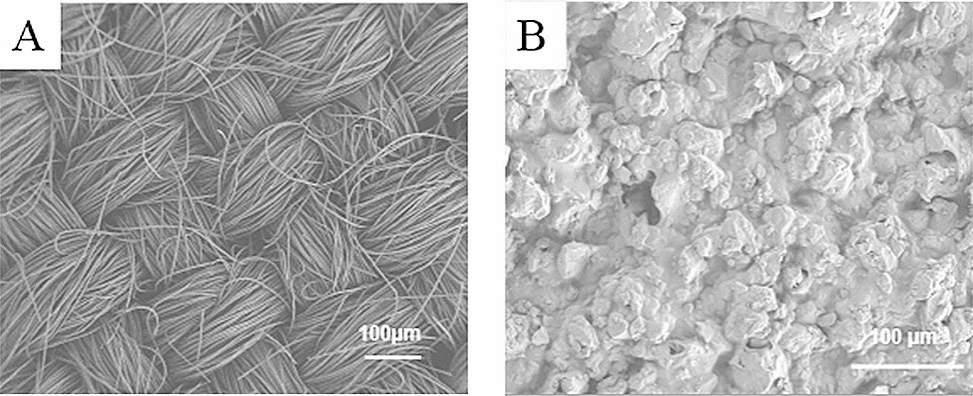
SEM images of anodes: **A** unmodified anode, **B** SAC/SA hydrogel modified anode

The physical form of the mixture gel on the electrode was observed using SEM. As shown in Fig. 1, compared to the carbon fiber surface of the blank carbon cloth anode (Fig. 1A), rougher surfaces, more granular lumps, and more grooves were observed on the SA modified anode (Fig. 1B). This morphological feature could provide more attachment sites for microorganisms, thereby facilitating an increased amount of attachment of microorganisms. This morphological feature could potentially enhance the availability of attachment sites for microorganisms, thereby facilitating an increased amount of microorganisms.

### The output voltages of air-cathode MFCs

**Fig. 2:**
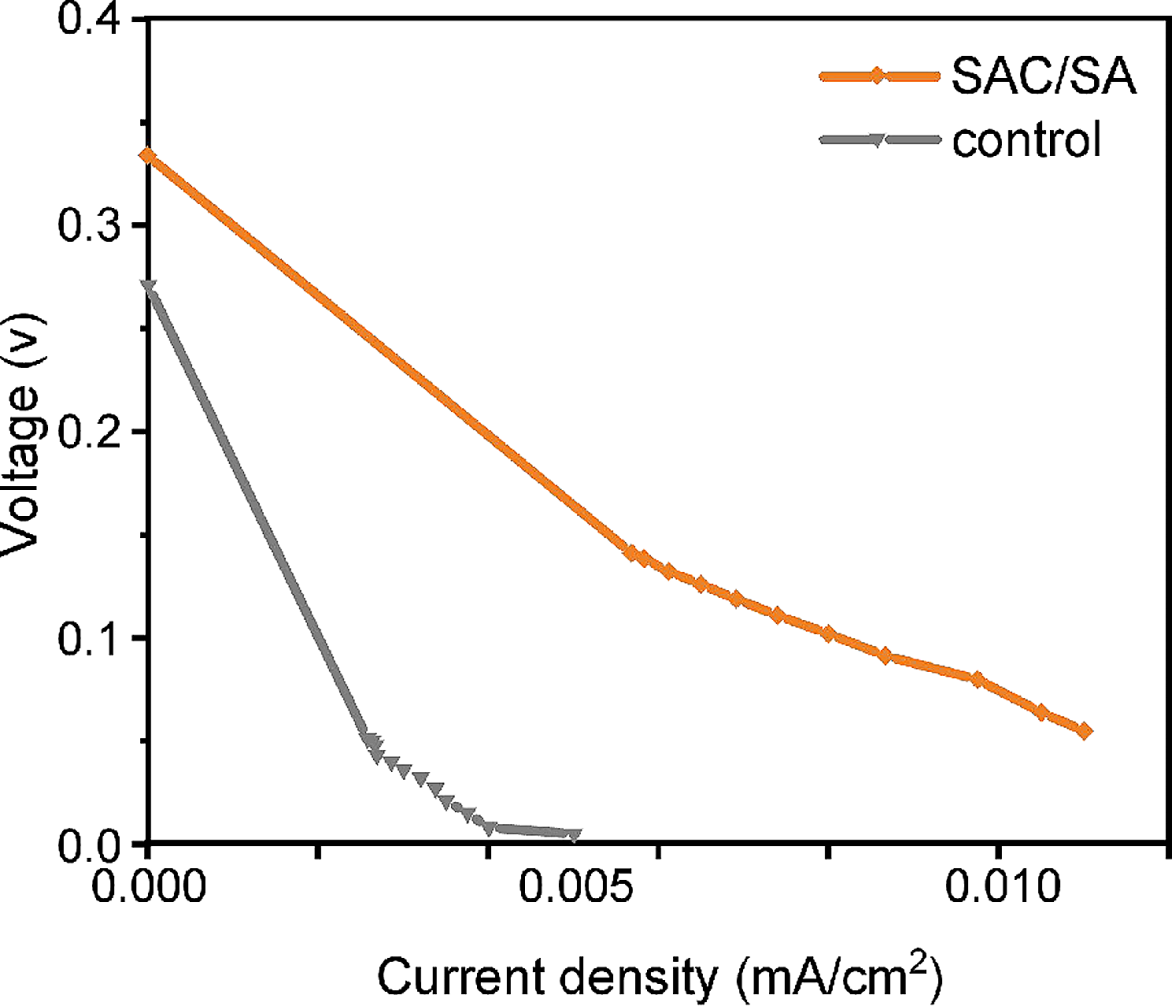
The output voltages for air-cathode MFCs with SAC/SA hydrogel modified and unmodified (control) anodes

As shown in Fig. 2, it was evident that the modification of SAC/SA hydrogel on the anodes significantly influences the start-up process of the MFCs. Its start-up time (6 h) was shorter than the control (16 h). These results indicated that the SAC/SA hydrogel modified anode could accelerate the start-up of the MFC. After 60 h, the voltage value of MFC modified with SAC/SA hydrogel anode reached steady state. The SAC/SA modified anode MFC generated a maximum voltage of about 0.028 V in the steady stage, which was 2.15 times that of the control (0.013 V) with blank carbon cloth anode.

### Polarization curves analysis

**Fig. 3:**
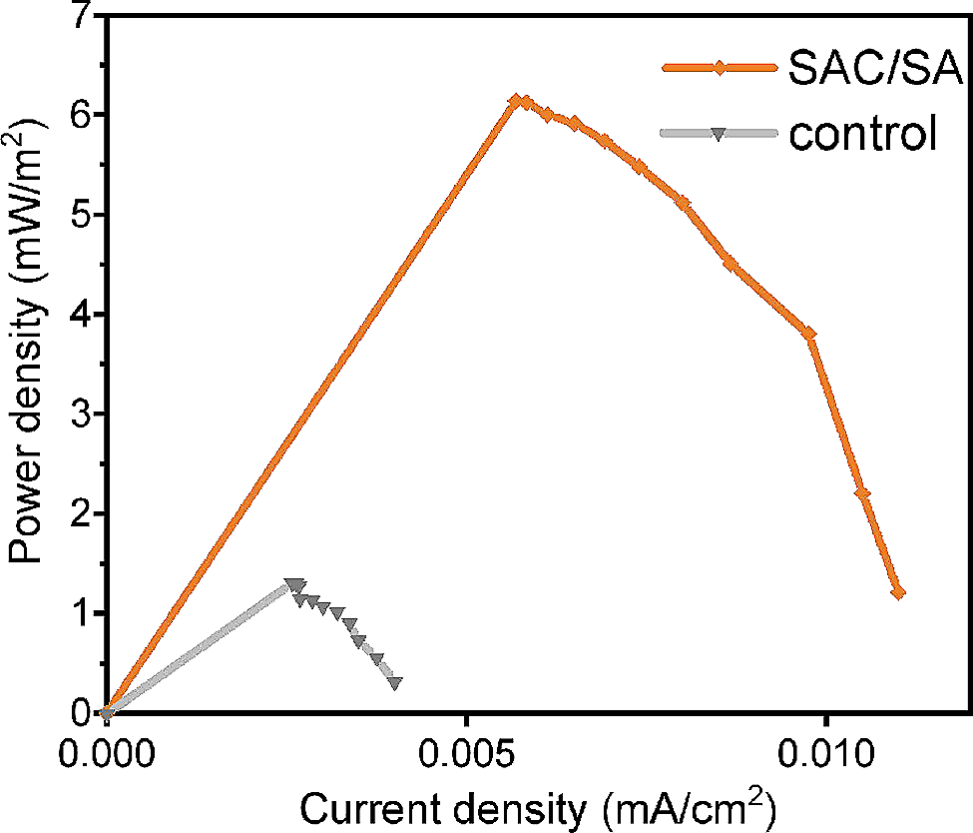
Polarization curves for MFCs with SAC/SA hydrogel modified and unmodified (control) anodes

The polarization curve was the characteristic curve of the MFC performance, which was obtained by the steady discharging method (Liang et al. 2007). The open circuit voltage (OCV, the current is 0, and the external resistance is infinite) of the MFC with control anode was 0.27 V (Fig. 3). The OCV of the MFC with modified anode was 0.33 V. With the change of the external resistance values, both the voltage and the current density changed. When the voltage was 0.021 V, the current density of the SAC modified anode was 0.01 mA/cm^2^, while the current density of the control anode was 0.0035 mA/cm^2^.

As one of the properties of a MFC, the lower the slope of the polarization curve, the smaller the resistance value of the MFC (Mohsin et al. 2020). Here, the slope of the polarization curve of the SAC modified anode was 26.9, which was lower than 27.4 of the blank carbon cloth anode MFC. Correspondingly, the internal resistance of the MFC with SAC/SA hydrogel modified anode was 9429 Ω, which was 18% lower than the blank carbon cloth anode (11560 Ω).

### Analysis of power density curves

**Fig. 4:**
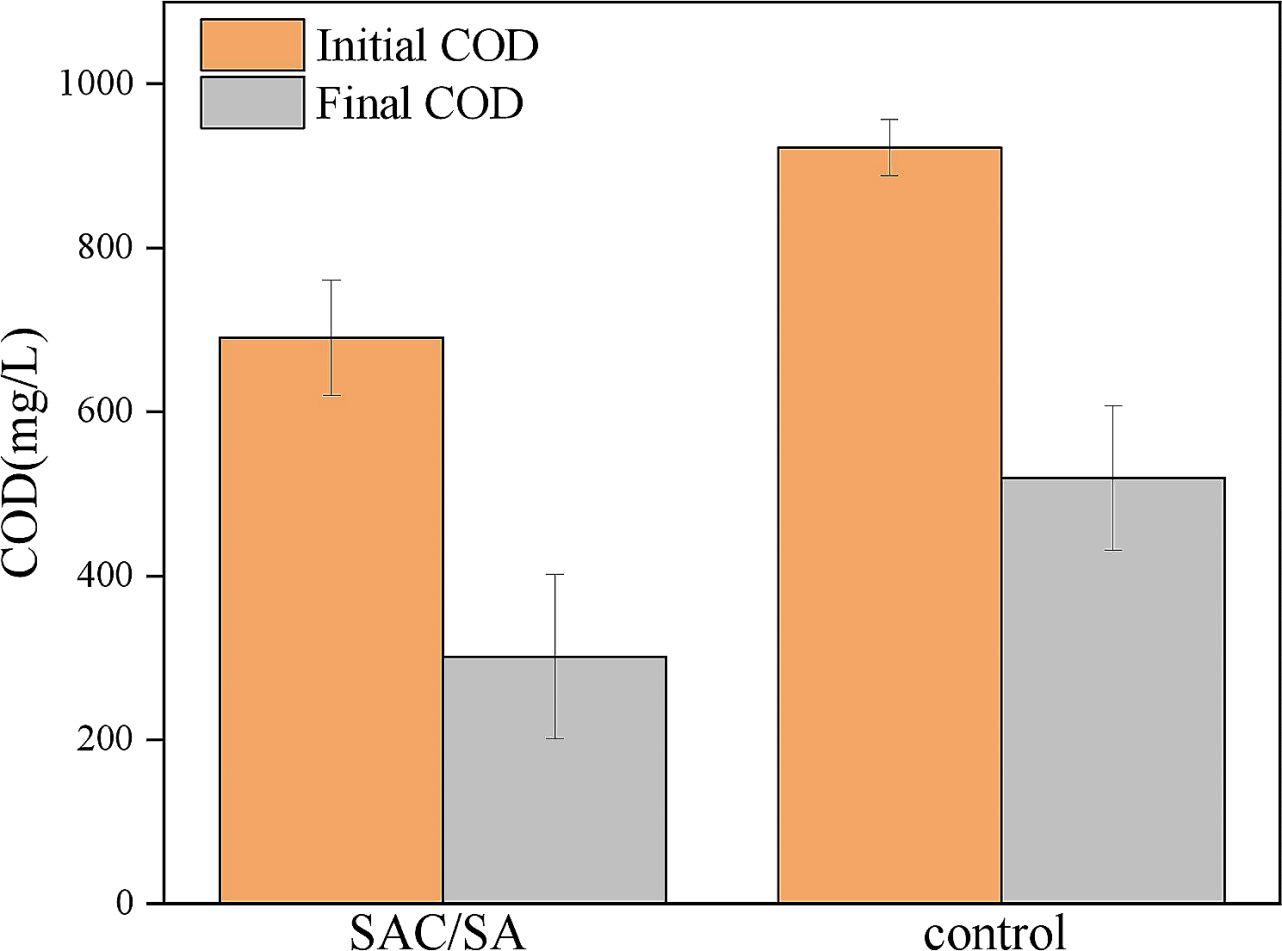
Power density curves for MFCs with SAC/SA hydrogel modified and unmodified (control) anodes

It was obviously that power density values of MFCs increased as the current density values (Fig. 4). When the external resistance was equal to the internal resistance of the MFC, the power density value reached the maximum (Fig. 4). Then the power density decreased as the current density increased because of the ohm loss and the increase of electrode overpotential (Wei 2010). The maximum power density values of the MFC with SAC/SA hydrogel modified anode and the control MFC were 6.14 and 1.32 mW/m^2^, respectively. The SAC/SA hydrogel modification increased the output power and improved the performance of the MFC. Its maximum power density value was 4.65 times that of the control MFC.

### COD removal efficiency and coulombic efficiency

**Fig. 5:**
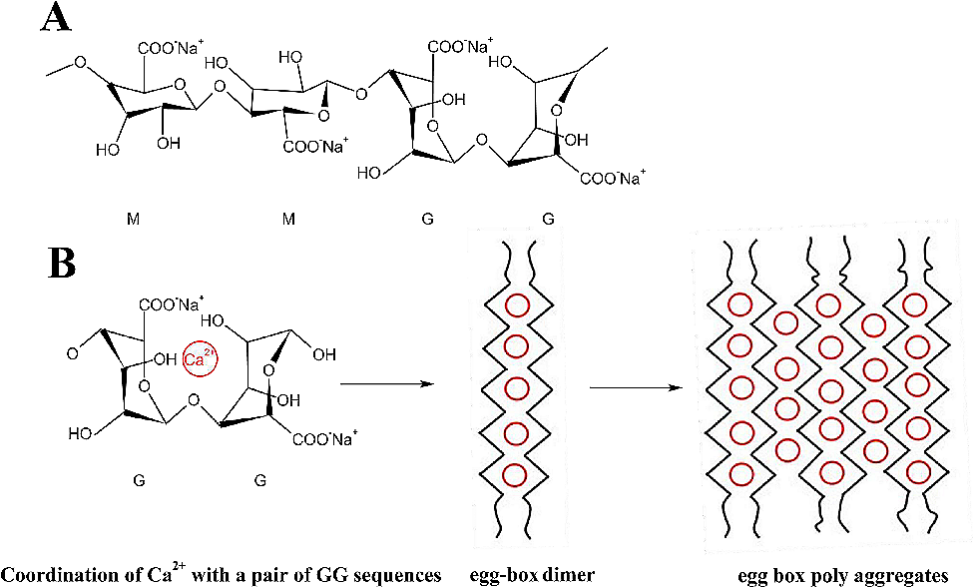
The COD removal efficiency in MFCs with SAC/SA hydrogel modified and unmodified (control) anodes

The COD removal efficiency and coulombic efficiency (CE) of the MFC anodes were further evaluated. The influent COD concentration was 690.8 mg/l (Fig. 5). After one cycle in the MFC with the SAC/SA hydrogel modified anode, the COD concentration reached 301.5 mg/l. The average removal efficiency was 56.36%, which was higher than 43.64% of MFC using blank carbon cloth anode. The CE of the SAC/SA hydrogel modified anode was calculated to be 17.65%, according to Eq. (1), which was higher than 8.65% of blank carbon cloth anode.

## Discussion

The use of SA as a binder to modify SAC on the electrode resulted in a significant improvement in the performance of the MFC. It was speculated that SAC played a major role in promoting electrical conduction during the operation of MFC. SAC was rich in mesopores (Zhang et al. 2022). The electrochemical performance of carbon materials was related to their pore structure (Lin et al. 2015; Liu 2017). The more mesopores and micropores of carbon materials, the better the electrochemical activity of carbon materials, the synergistic effect of the two would further enhance the electrochemical activity of MFCs (Ferrero et al. 2016; Liu 2017). The pore structure could effectively increase the specific surface area for extracellular electron transfer (Lin et al. 2015). SAC referred to activated carbon with a specific surface area greater than 2000 m^2^/g, which had a high pore volume (> 2 cm^3^/g) due to its mesopores and micropores (Zhang et al. 2022). The micropores of SAC could increase the specific surface area of ions and form an effective ion-electroadsorption electric double layer (Chen et al. 2018). The mesopores of SAC could reduce the ion diffusion resistance in the channel and improve the availability of the ion adsorption surface area (Zhang et al. 2022). Therefore, SAC had a higher capacitance (Zhang et al. 2022), which could facilitate the electron transfer on the electrodes of the MFCs.

In addition, SA, as an adhesive provided a good support for the adhesion of SAC (Lacoste et al. 2018). Because alginate was a natural polysaccharide composed of different proportions of β-d-1,4-mannuronic acid (M) blocks and α-l-1,4-guluronic acid (G) blocks (Salomonsen et al. 2008), in which irregular MM, GG, and MG block patterns could be formed (Fig. 5A). There was a space steric hindrance around the carboxylic acid group (Salomonsen et al. 2008). Thus, M blocks formed linear domains and G blocks formed bent or distorted regions resulting in a more rigid structure (Hecht and Srebnik 2016). The most conventional binder (polyvinylidene fluoride, PVDF) used for the batteries was attached to particles via weak van der waals forces only, and failed to accommodate large changes in spacing between the particles (Magasinski et al. 2010). SA contained hydroxyl groups, and stable hydrogen bonds could be formed between these hydroxyl groups, which could strengthen the adhesion between molecules and formed a stable hydrogel. It firmly binded the SAC, thereby more fully assisting the SAC to function as an electrical conductor, and finally promoting the improvement of the electrochemical performance of the MFC. In addition, it also had higher stability compared to traditional PVDF adhesives (Park et al. 2011).

**Fig. 6:**
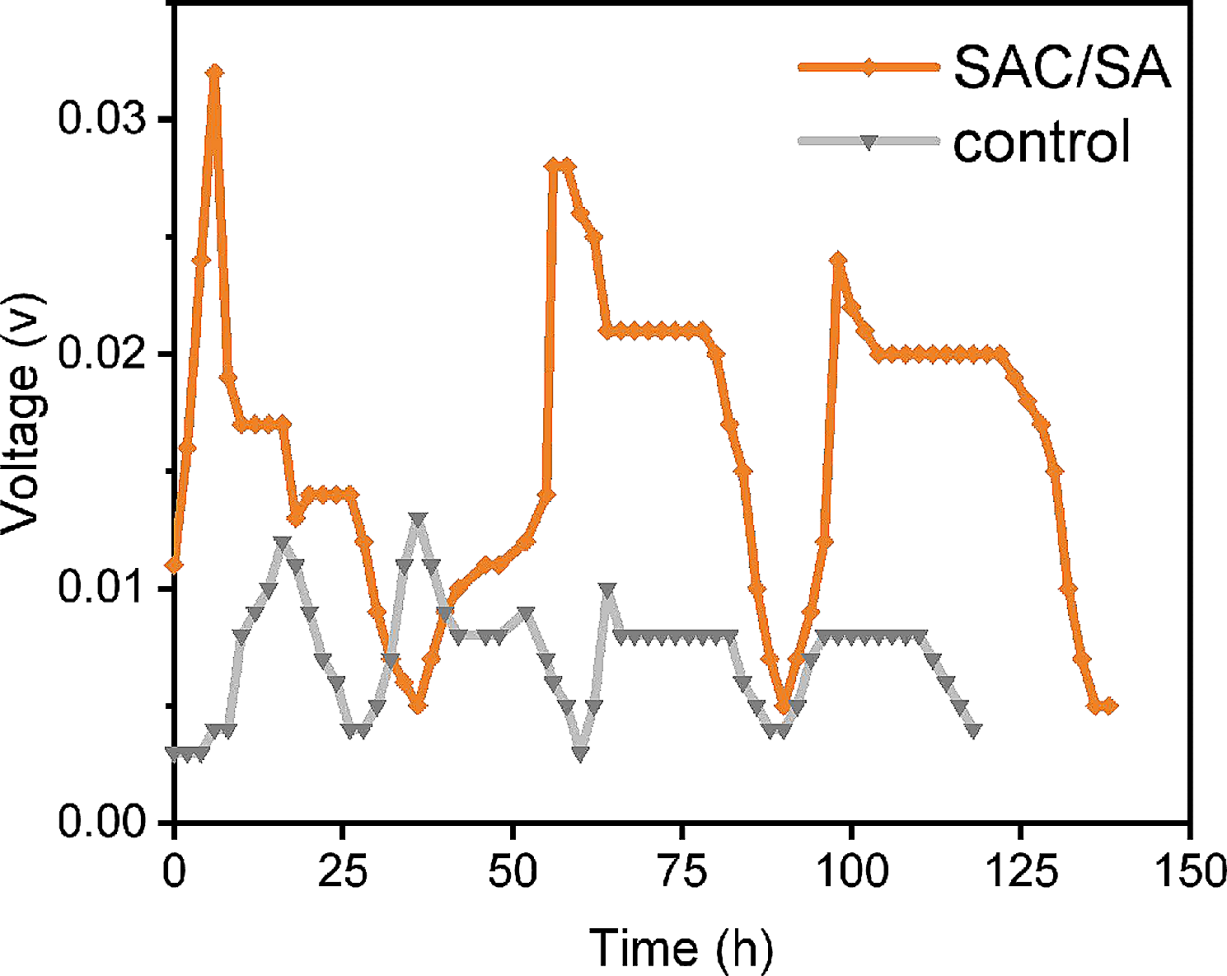
**A** Structure of SA; **B** Schematic diagram of egg-box connection structure in alginate/calcium gels. The open circles represented Ca^2+^ ions

The structure of SA resulted in a larger attachment space. After Ca^2+^ was added to alginate, the GG blocks binded to the Ca^2+^ ion to form an egg-box dimer (Fig. 6B) (Grant et al. 1973). The GG blocks along the polymer backbone (Fig. 6B) started to form viscous ionic hydrogels (Qin 2008). The binding of ploy-G residues to Ca^2+^ formed a stable “egg-box” structure with more kinks and deeper nests (Hecht and Srebnik 2016). This structure of SA gel could further increase the space for more SAC and microorganisms attaching to the anodes (Zhang et al. 2011). The large specific surface area increased the reaction area and reduced the internal resistance of the MFC. The internal resistance was closely related to the output power of the MFC (Zhang and Liu 2010). Lowering the internal resistance could greatly improve the power output (Wen et al. 2010). This resulted in an increase in the maximum output power.

In summary, the use of SA hydrogel mixed with SAC to modify the anode was a simple and practical modification method. It enabled MFC to have faster electron transfer efficiency and lower internal resistance, increased the output voltage and power, and improved the COD removal efficiency and MFC performance. Our work provided details for future improvement in algal bioresources as binders. It could provide a reference for the follow-up research to improve the performance of MFC.

## Data Availability

The datasets used and/or analyzed during the current study are available from the corresponding author on reasonable request.
